# Impact of exposure to cooking fuels on stillbirths, perinatal, very early and late neonatal mortality - a multicenter prospective cohort study in rural communities in India, Pakistan, Kenya, Zambia and Guatemala

**DOI:** 10.1186/s40748-015-0019-0

**Published:** 2015-07-21

**Authors:** Archana B. Patel, Sreelatha Meleth, Omrana Pasha, Shivaprasad S. Goudar, Fabian Esamai, Ana L. Garces, Elwyn Chomba, Elizabeth M. McClure, Linda L. Wright, Marion Koso-Thomas, Janet L. Moore, Sarah Saleem, Edward A. Liechty, Robert L. Goldenberg, Richard J. Derman, K. Michael Hambidge, Waldemar A. Carlo, Patricia L. Hibberd

**Affiliations:** Lata Medical Research Foundation, Nagpur, Maharashtra 440022 India; RTI International, Research Triangle Park, North Carolina, 27709 USA; Department of Community Health Sciences & Family Medicine, Aga Khan University, Karachi, Pakistan; KLE University’s JN Medical College, Belgaum, Karnataka India; Moi University School of Medicine, Eldoret, Kenya; IMSALUD, San Carlos University, Guatemala City, Guatemala; University Teaching Hospital, Lusaka, Zambia; Center for Research of Mothers and Children, NIH, Rockville, MD 20852 USA; Department of Pediatrics, Indiana University School of Medicine, Indianapolis, IN 46202 USA; Department of Obstetrics/Gynecology, Columbia University, New York, NY 10032 USA; Department of OB-GYN, Christiana Care, Newark, DE 19718 USA; Department of Pediatrics, University of Colorado, Aurora, CO 80045 USA; Department of Pediatrics, University of Alabama at Birmingham, Birmingham, AL 35233 USA; Division of Global Health, Department of Pediatrics, Massachusetts General Hospital, Boston, MA 02114 USA

**Keywords:** Perinatal, Neonatal, Mortality, Cooking fuels, Household air pollution

## Abstract

**Background:**

Consequences of exposure to household air pollution (HAP) from biomass fuels used for cooking on neonatal deaths and stillbirths is poorly understood. In a large multi-country observational study, we examined whether exposure to HAP was associated with perinatal mortality (stillbirths from gestation week 20 and deaths through day 7 of life) as well as when the deaths occurred (macerated, non-macerated stillbirths, very early neonatal mortality (day 0–2) and later neonatal mortality (day 3–28).

Questions addressing household fuel use were asked at pregnancy, delivery, and neonatal follow-up visits in a prospective cohort study of pregnant women in rural communities in five low and lower middle income countries participating in the Global Network for Women and Children’s Health’s Maternal and Newborn Health Registry. The study was conducted between May 2011 and October 2012. Polluting fuels included kerosene, charcoal, coal, wood, straw, crop waste and dung. Clean fuels included electricity, liquefied petroleum gas (LPG), natural gas and biogas.

**Results:**

We studied the outcomes of 65,912 singleton pregnancies, 18 % from households using clean fuels (59 % LPG) and 82 % from households using polluting fuels (86 % wood). Compared to households cooking with clean fuels, there was an increased risk of perinatal mortality among households using polluting fuels (adjusted relative risk (aRR) 1.44, 95 % confidence interval (CI) 1.30-1.61). Exposure to HAP increased the risk of having a macerated stillbirth (adjusted odds ratio (aOR) 1.66, 95%CI 1.23-2.25), non-macerated stillbirth (aOR 1.43, 95 % CI 1.15-1.85) and very early neonatal mortality (aOR 1.82, 95 % CI 1.47-2.22).

**Conclusions:**

Perinatal mortality was associated with exposure to HAP from week 20 of pregnancy through at least day 2 of life. Since pregnancy losses before labor and delivery are difficult to track, the effect of exposure to polluting fuels on global perinatal mortality may have previously been underestimated.

**Trial registration:**

ClinicalTrials.gov NCT01073475

## Background

As progress continues to be made toward Millennium Development Goal #4 (MDG4), attention increasingly focuses on causes of childhood mortality that have been the most resistant to improvement – particularly neonatal mortality (through day 28 of life) [1–4]. Reducing stillbirths (after week 20 of pregnancy and particularly intrapartum [3, 5]) is not addressed in MDG#4 (which focuses only on babies born alive) but the importance of reducing the burden of stillbirths, many of which may be resuscitatable at birth has been increasingly recognized [6].

Solid fuels and kerosene are used for cooking, heating and lighting by one third of the world’s population [7]. Inefficient burning of these fuels results in household air pollution (HAP) that includes particulate matter and toxic chemicals, such as hydrocarbons, oxygenated organic compounds, free radicals and carbon monoxide [8]. HAP is the fourth leading risk factor for the global burden of disease, accounting for 3.5 million premature deaths in adults and children annually [7, 9]. HAP is a recognized risk factor for childhood pneumonia [10] and preterm birth [11], but the role of exposure to HAP on other pregnancy and neonatal outcomes is less clear due to concerns about the quality of evidence in the available observational studies [10]. This information is important as international governments are rolling out improved cookstoves that continue to use solid fuels without evidence on potential perinatal and other health benefits [12]. In addition, while there is a biologic basis for the effects of HAP on the developing fetus, neonate and young infant based on the similar pollutants in tobacco smoke (active and passive smoke exposure) [13–15], it is also unclear whether the effects of tobacco smoking and HAP are additive, synergistic or whether there is no interaction because the effect of one of the exposures (e.g., HAP) overwhelms the other (e.g., tobacco smoke).

The *Eunice Kennedy Shriver* National Institute of Child Health and Human Development’s (NICHD’s) Global Network (GN) for Women and Children’s Health Research supports a Maternal and Newborn Health (MNH) Registry of pregnant women and their babies living in rural communities in low and lower middle income countries. The Registry has focused on documentation of fetal loss after week 20 of pregnancy, accurate and timely measurement of birth, birth weight and early and late neonatal outcomes [16]. It provides an ideal population to address unanswered questions about risk factors for perinatal mortality as well as the timing of fetal loss or neonatal death. Thus our primary objective was to examine whether HAP from cooking with biomass fuels was associated with perinatal mortality (stillbirths from gestation week 20 and deaths through day 7 of life). Secondary objectives were to examine whether HAP exposure was a risk factor for macerated, non-macerated stillbirths, very early neonatal mortality (day 0–2 of life) and mortality from day 3–28 of life. We also address recent issues raised about the use of kerosene as a polluting fuel because of concerns that it has previously inappropriately considered a clean fuel [7].

## Methods

### Ethics statement

The MNH Registry is an ongoing prospective multicentre cohort study of pregnant women and their babies in 100 rural communities located in Guatemala, 2 states in India, Kenya, Pakistan and Zambia. Pregnant women are recruited as early as possible during pregnancy and followed through day 42 post-partum to obtain details about the pregnancy, labor and delivery and the health of the mother and infant. The study was reviewed and approved at all of the involved institutions’ ethics review committees at: The Lata Medical Research Foundation, Nagpur, Maharashtra, India; Aga Khan University, Karachi, Pakistan; JN Medical College, Belgaum, Karnataka, India; Moi University School of Medicine, Eldoret, Kenya; IMSALUD, San Carlos University, Guatemala City, Guatemala; University Teaching Hospital, Lusaka, Zambia; Indiana University School of Medicine, Indianapolis, Indiana; Columbia University, New York, New York; Christiana Care, Newark, Delaware; University of Colorado, Aurora, Colorado; University of Alabama at Birmingham, Birmingham, Alabama; Partners IRB, Massachusetts General Hospital, Boston, Massachusetts and RTI International, Research Triangle Park, North Carolina. The study was registered at ClinicalTrials.gov (NCT01073475). A Data Monitoring Committee appointed by NICHD reviewed the registry data on at least an annual basis.

Pregnant women intending to deliver in the study communities or affiliated hospitals were informed about the study and invited to participate in the MNH Registry. Those who consented signed the IRB approved informed consent form.

### Study design, setting and participants

**Fig. 1:**
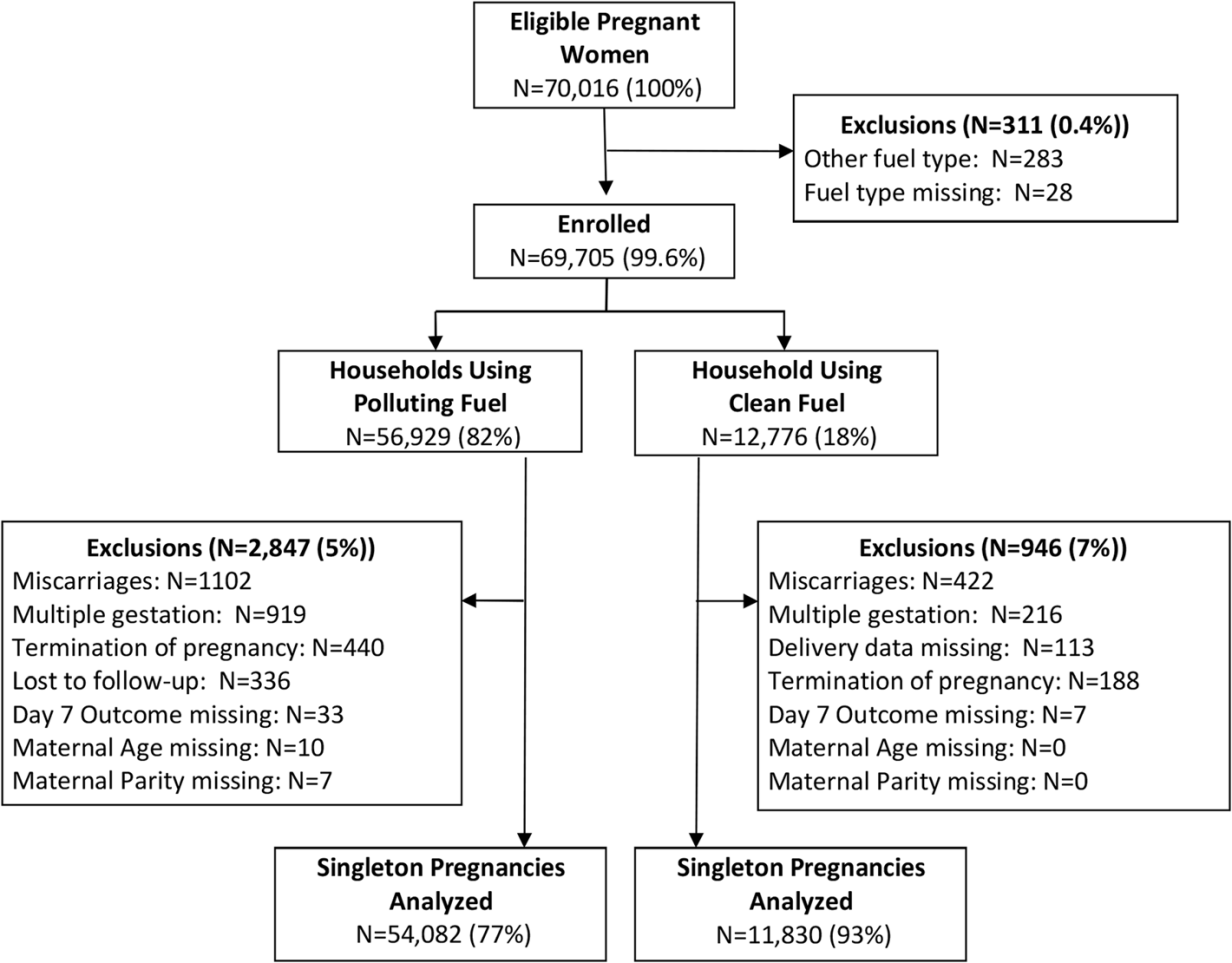
Study flow diagram

We included pregnant women enrolled in the MNH Registry. We excluded women from households for which there was incomplete information on type of cooking fuel used in the household, multiple gestations, as well as women who had a medical termination of pregnancy or miscarriage before week 20 of pregnancy, and women who had incomplete information on maternal parity or age, or were lost to follow-up (Fig. 1).

Information is obtained at three time points in the registry. On enrolment (before the 20^th^ week of gestation), information on the date of last menstrual period, estimated delivery date, age, education, parity, and status of last child is collected. Within 7 days of delivery, information is collected on prenatal care, birth preparedness, complications occurring during pregnancy, details of labor and delivery, including place, mode of delivery, provider, actual birth weight obtained at the time of birth, status of the mother and newborn following delivery, referrals, and treatment provided to the mother and newborn at referral facilities. Interval maternal and newborn health and status is assessed 42 days after birth. Birth weight is recorded for all babies (live born and still births) using locally available scales, calibrated per the local facilities. All study area birth attendants are trained to use and record accurate birth weights as described previously [16].

In May 2011, questions adapted from the Demographic Health Survey’s (DHS) Household questionnaire, version 6 [17] on the type of fuel, location used for cooking and tobacco smoking in the household were added to the MNH Registry Questionnaire during the day 42 post-partum visit.

### Study variables

#### Exposures

Households using only electricity, liquefied petroleum gas, natural gas and biogas for cooking in their primary and secondary home (if they moved to a second location during pregnancy) were classified as homes using clean fuels. Households using all other fuels for cooking (kerosene, charcoal, coal, wood, straw, crop waste and dung) were classified as homes using polluting fuels. The location of cooking in the house was classified as in the house (separate kitchen or no separate kitchen), in a separate building or outside.

#### Outcomes

##### Primary

Perinatal mortality – fetal loss after week 20 of pregnancy through day 7 of life (macerated stillbirths + non-macerated stillbirths + early neonatal mortality (NMR_0-7))/all pregnancies.

##### Secondary

(i) Macerated stillbirths/all pregnancies
(ii) Non-macerated stillbirths/all pregnancies
(iii) Very early neonatal mortality (NMR_0-2) through day 2 of life/all live births
(iv) Later neonatal mortality (NMR_3-28) from day 3–28 of life/all live births

A stillbirth was defined as birth of a baby after week 20 of gestation that had no signs of life at birth (no gasping, breathing, heart beat or movement). Stillbirths were further classified as macerated (death presumed before onset of labor, based on presence of discoloration and peeling of the skin leaving areas of raw tissue, an unusually soft skull, a dark red or black stained umbilical cord or darkly stained amniotic fluid) vs. non-macerated stillbirth (presumed intrapartum death and no signs of maceration).

#### Covariates

We collected data on the following covariates: maternal age (<20, ≥20); education (no formal education, any formal education); parity (0, 1–2, ≥ 3); gestational age (preterm (<37 weeks) or term (≥37 weeks) as assessed by last menstrual period, clinical examination, ultrasound, or other method); delivery location (hospital, clinic, home or other location); birth weight using available local scales; infant gender; and household tobacco use (anyone in the household smoking inside the house at least daily, less than daily smoking or no smokers in the household).

#### Data source

All study data were obtained by trained interviewers who recorded the response on case report forms. The interviewers were unaware of the study hypotheses.

#### Statistical considerations

##### Sample size

The sample size calculations were based on the assumption that exposure to HAP would increase the risk of perinatal mortality by approximately 1.23 based on the lower 95 % confidence interval of a previously reported odds ratios from a meta-analysis for stillbirths (there are no previously published data for perinatal mortality) and HAP of 1.5 (95 % CI 1.23, 1.85) [18]. Sample size was calculated conservatively and based on the lower level of the reported 95 % CI, although kerosene was classified as a clean fuel in the meta-analysis, so this estimate is conservative. Based on the MNH Registry data for 2010 [16], we assumed a baseline perinatal mortality rate of 32/1000 in the unexposed group. To detect an OR 1.23 (PMR of 37/1000 or greater in the exposed group), significant at alpha = 0.05 (2 sided), with 80 % power, we estimated that we would need to collect outcome data on 61,530 singleton births.

##### Methods

We first estimated population averaged effects of HAP on perinatal mortality using generalized estimating equations (GEE) to control for correlations within clusters. We fitted a modified Poisson regression model with a sandwich error estimation. All relative risks were adjusted for site due to the variability across the sites in the Global Network. Bivariate associations between covariates such as mother’s age, mother’s education, parity, ante-natal care, birth attendants at the delivery and mortality were evaluated by fitting a regression model that controlled for site and had mortality as the outcome and the covariate of interest as the predictor. We elected not to include low birth weight in the model as a covariate because it may be an intermediate step in the causal pathway between exposure to HAP and perinatal mortality [19–21]. All covariates with significant RRs were included in a final model that had PMR as the outcome and HAP as a predictor.

Since exposure to HAP would have differential effects on the fetus during pregnancy through the first month of life, particularly on macerated stillbirths, non-macerated stillbirths, very neonatal mortality through day 2 of life (NMR_0-2) and later neonatal mortality from day 3–28 of life (NMR_3-28), we also modeled the data using multinomial logistic regression with a 5 level nominal outcome (macerated stillbirth, non-macerated stillbirth, NMR_0-2, NMR_3-28, alive after day 28). The model included exposure to HAP as the predictor and controlled for the same covariates as above. Low birth weight was excluded as explained above. We used the clustered bootstrap method [22] to estimate the variance of the estimates and create 95 % confidence intervals.

## Results

**Fig. 2:**
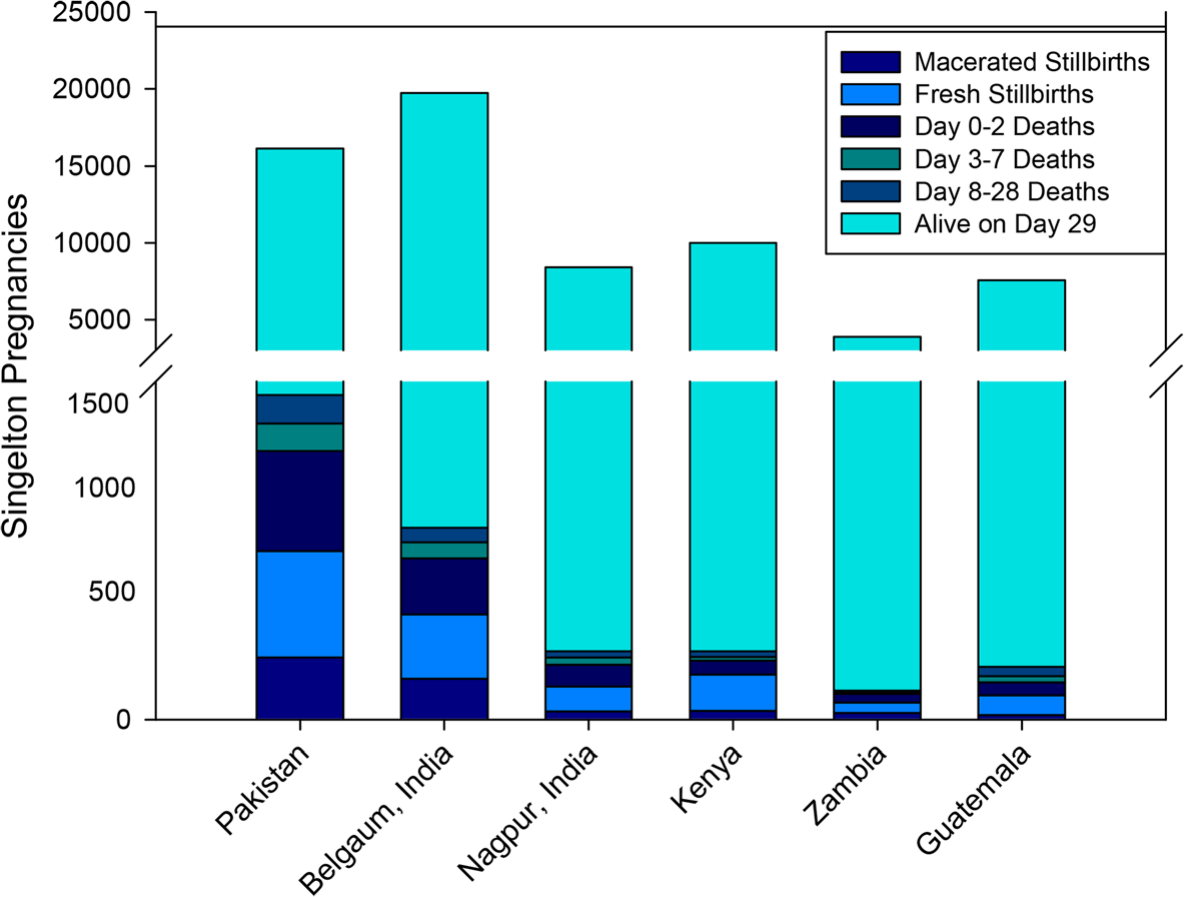
Pregnancy outcomes by global network site

**Fig. 3:**
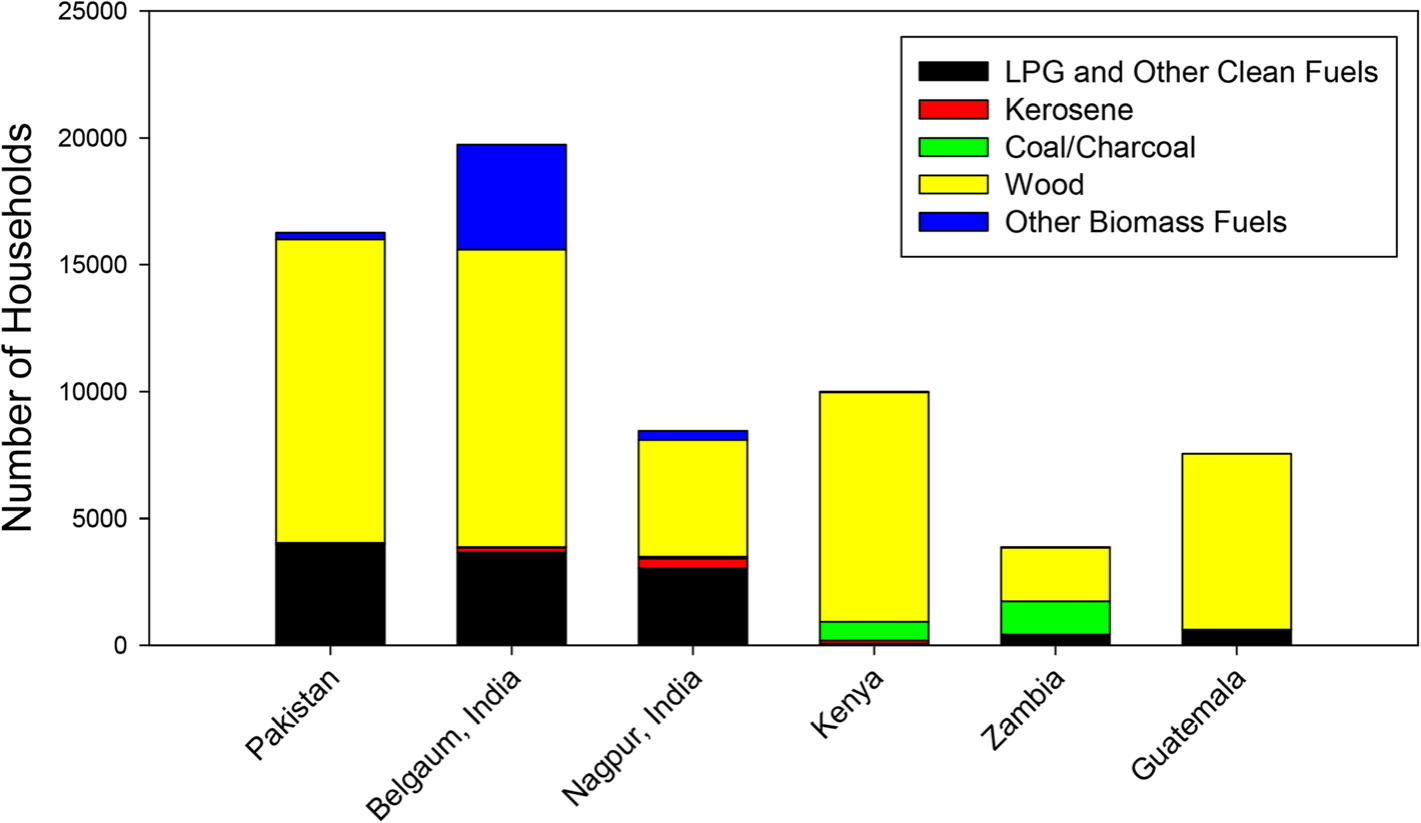
Fuel use by global network site

Between May 2011 and Oct 2012, we studied 65,912 pregnant women (Fig. 1). Mortality outcomes were available for 65,701 births (99.7 %). There were 1,740 stillbirths (577 macerated and 1,163 non-macerated stillbirths) and 63,961 live births, of which 950 died on or before the second day of life, 275 died between the 3^rd^ and 7^th^ day of life, and 295 died between the 8^th^ and 28^th^ day of life). The distribution of the pregnancy outcomes by geographic location is shown in Fig. 2. Table 1 shows the demographic characteristics of the pregnant women, births and the households including details of the fuels used for cooking. A total of 54,082 (82 %) pregnancies occurred in households using polluting fuel and 11,830 (18 %) in households using clean fuels. The distribution of pregnancies by types of fuel use and geographic location is shown in Fig. 3. LPG was the predominant type of clean fuel (59 %) followed by natural gas (33 %) and wood was the predominant type of polluting fuel (86 %).

**Table 1:** Demographic characteristics of the study subjects and households

Characteristic	Pakistan	Belgaum, India	Nagpur, India	Kenya	Zambia	Guatemala	Total
Maternal age	16,235	19,728	8,443	9,973	3,876	7,554	65,809
<20	626 (3.9)	1,835 (9.3)	168 (2.0)	2,183 (21.9)	984 (25.4)	1,276 (16.9)	7,072 (10.7)
≥20	15,609 (96.1)	17,893 (90.7)	8,275 (98.0)	7,790 (78.1)	2,892 (74.6)	6,278 (83.1)	58,737 (89.3)
Maternal education	16,239	19,611	8,439	9,974	3,847	7,555	65,665
No formal schooling	13,595 (83.7)	4,464 (22.8)	216 (2.6)	295 (3.0)	422 (11.0)	1,610 (21.3)	20,602 (31.4)
Primary	1,163 (7.2)	5,998 (30.6)	1,414 (16.8)	7,100 (71.2)	2,084 (54.2)	4,769 (63.1)	22,528 (34.3)
Secondary	900 (5.5)	7,278 (37.1)	5,068 (60.1)	2,216 (22.2)	1,275 (33.1)	1,126 (14.9)	17,863 (27.2)
University+	581 (3.6)	1,871 (9.5)	1,741 (20.6)	363 (3.6)	66 (1.7)	50 (0.7)	4,672 (7.1)
Parity	16,238	19,384	8,446	9,976	3,876	7,554	65,474
0	3,412 (21.0)	8,321 (42.9)	4,119 (48.8)	2,498 (25.0)	1,036 (26.7)	2,056 (27.2)	21,442 (32.7)
1-2	5,325 (32.8)	9,746 (50.3)	4,110 (48.7)	4,000 (40.1)	1,455 (37.5)	2,664 (35.3)	27,300 (41.7)
≥3	7,501 (46.2)	1,317 (6.8)	217 (2.6)	3,478 (34.9)	1,385 (35.7)	2,834 (37.5)	16,732 (25.6)
Antenatal care	16,121	19,696	8,426	9,981	3,874	7,529	65,627
Any	14,385 (89.2)	19,689 (100.0)	8,425 (100.0)	9,794 (98.1)	3,856 (99.5)	7,349 (97.6)	63,498 (96.8)
None	1,736 (10.8)	7 (0.0)	1 (0.0)	187 (1.9)	18 (0.5)	180 (2.4)	2,129 (3.2)
Number of antenatal visits	13,557	16,004	8,420	9,794	787	7,349	55,911
1	2,889 (21.3)	954 (6.0)	108 (1.3)	468 (4.8)	124 (15.8)	365 (5.0)	4,908 (8.8)
2	4,617 (34.1)	1,268 (7.9)	84 (1.0)	1,674 (17.1)	148 (18.8)	735 (10.0)	8,526 (15.2)
3	3,359 (24.8)	4,436 (27.7)	502 (6.0)	3,649 (37.3)	269 (34.2)	1,386 (18.9)	13,601 (24.3)
≥4	2,692 (19.9)	9,346 (58.4)	7,726 (91.8)	4,003 (40.9)	246 (31.3)	4,863 (66.2)	28,876 (51.6)
Delivery attendant	16,266	19,755	8,444	9,991	3,881	7,555	65,892
Physician	4,066 (25.0)	11,761 (59.5)	4,963 (58.8)	200 (2.0)	83 (2.1)	2,785 (36.9)	23,858 (36.2)
Nurse/Midwife	4,312 (26.5)	7,055 (35.7)	3,249 (38.5)	3,944 (39.5)	2,067 (53.3)	197 (2.6)	20,824 (31.6)
No skilled birth attendant	7,888 (48.5)	939 (4.8)	232 (2.7)	5,847 (58.5)	1,731 (44.6)	4,573 (60.5)	21,210 (32.2)
Delivery location	16,275	19,755	8,445	9,989	3,881	7,555	65,900
Facility	8,624 (53.0)	18,673 (94.5)	8,186 (96.9)	4,012 (40.2)	2,340 (60.3)	2,960 (39.2)	44,795 (68.0)
Home/Other	7,651 (47.0)	1,082 (5.5)	259 (3.1)	5,977 (59.8)	1,541 (39.7)	4,595 (60.8)	21,105 (32.0)
Infant gender	16,135	19,722	8,390	9,983	3,873	7,551	65,654
Male	8,472 (52.5)	10,335 (52.4)	4,353 (51.9)	5,069 (50.8)	2,038 (52.6)	3,816 (50.5)	34,083 (51.9)
Female	7,663 (47.5)	9,387 (47.6)	4,037 (48.1)	4,914 (49.2)	1,835 (47.4)	3,735 (49.5)	31,571 (48.1)
Fuel used for cooking	16,276	19,755	8,450	9,995	3,881	7,555	65,912
Electricity	50 (0.3)	69 (0.3)	83 (1.0)	6 (0.1)	434 (11.2)	3 (0.0)	645 (1.0)
Liquified petroleum gas	51 (0.3)	3,503 (17.7)	2,837 (33.6)	15 (0.2)	0 (0.0)	576 (7.6)	6,982 (10.6)
Natural gas	3,861 (23.7)	3 (0.0)	16 (0.2)	29 (0.3)	2 (0.1)	27 (0.4)	3,938 (6.0)
Biogas	11 (0.1)	95 (0.5)	123 (1.5)	23 (0.2)	0 (0.0)	5 (0.1)	257 (0.4)
Kerosene	61 (0.4)	198 (1.0)	370 (4.4)	132 (1.3)	6 (0.2)	16 (0.2)	783 (1.2)
Coal	13 (0.1)	3 (0.0)	61 (0.7)	4 (0.0)	0 (0.0)	3 (0.0)	84 (0.1)
Charcoal	3 (0.0)	2 (0.0)	15 (0.2)	733 (7.3)	1,306 (33.7)	0 (0.0)	2,059 (3.1)
Wood	11,968 (73.5)	11,735 (59.4)	4,589 (54.3)	9,033 (90.4)	2,120 (54.6)	6,925 (91.7)	46,370 (70.4)
Straw, etc.	84 (0.5)	369 (1.9)	98 (1.2)	18 (0.2)	1 (0.0)	0 (0.0)	570 (0.9)
Agricultural crop	19 (0.1)	2,398 (12.1)	179 (2.1)	2 (0.0)	0 (0.0)	0 (0.0)	2,598 (3.9)
Animal dung	154 (0.9)	1,380 (7.0)	78 (0.9)	0 (0.0)	0 (0.0)	0 (0.0)	1,612 (2.4)
No food cooked in household/other	1 (0.0)	0 (0.0)	1 (0.0)	0 (0.0)	12 (0.3)	0 (0.0)	14 (0.0)
Smoking in primary household	16,272	19,667	8,439	9,993	3,858	7,555	65,784
Daily	5,967 (36.7)	3,446 (17.5)	2,297 (27.2)	905 (9.1)	1,065 (27.6)	123 (1.6)	13,803 (21.0)
Less than daily	259 (1.6)	982 (5.0)	1,681 (19.9)	1,537 (15.4)	232 (6.0)	370 (4.9)	5,061 (7.7)
No smoking	10,046 (61.7)	15,239 (77.5)	4,461 (52.9)	7,551 (75.6)	2,561 (66.4)	7,062 (93.5)	46,920 (71.3)
Cooking location for primary household	16,272	19,666	8,430	9,992	3,858	7,555	65,773
In the house	4,010 (24.6)	19,280 (98.0)	7,768 (92.1)	2,693 (27.0)	860 (22.3)	956 (12.7)	35,567 (54.1)
In a separate building	9,149 (56.2)	284 (1.4)	559 (6.6)	6,930 (69.4)	2,462 (63.8)	3,424 (45.3)	22,808 (34.7)
Outdoors	3,110 (19.1)	100 (0.5)	102 (1.2)	367 (3.7)	519 (13.5)	3,172 (42.0)	7,370 (11.2)
Other	3 (0.0)	2 (0.0)	1 (0.0)	2 (0.0)	17 (0.4)	3 (0.0)	28 (0.0)

The overall perinatal mortality rate was 48/1,000 pregnancies > 20 weeks gestation, ranging from 25/1,000 pregnancies in Guatemala to 90/1,000 pregnancies in Pakistan. Table 2 shows the adjusted and unadjusted relative risks (RR) for exposure to HAP and covariates that were estimated using GEE in a Poisson regression model. The adjusted relative risk for perinatal mortality among babies whose mothers were exposed to HAP vs. clean fuels during pregnancy was 1.44 (95 % CI 1.30, 1.61). Risk factors for perinatal mortality in the multivariate analysis, also adjusted for Global Network site, included cooking with polluting fuel, lack of maternal education (no formal schooling), nulliparity and 3 or more prior births, no antenatal care, and male gender. Delivery by nurse or midwife or delivery unattended by a trained birth attendant was associated with lower perinatal mortality compared with delivery by a physician. Presence of anyone in the household who smoked on a daily basis was not associated with perinatal mortality.

**Table 2:** Risk factors for perinatal mortality

Characteristic	Perinatal mortality (through day 7 of life)	Alive on day 8 of life	Relative risk adjusted for global network site and community (95 % confidence interval)	Multivariate analysis - adjusted relative risk (95 % confidence interval)
	N = 3,176 n (%)	N = 62,736 n (%)		
HAP exposure	3,176	62,736		
Polluting fuel	2,683 (84)	51,399 (82)	1.36 (1.25, 1.49)	1.44 (1.30, 1.61)
Clean fuel	493 (16)	11,337 (18)	1.00	1.00
Maternal age	3,172	62,637		
<20	294 (9)	6,778 (11)	1.14 (1.01, 1.29)	1.03 (0.90, 1.18)
≥20	2,878 (91)	55,859 (89)	1.00	1.00
Maternal education	3,168	62,497		
No formal schooling	1,589 (50)	19,013 (30)	1.27 (1.12, 1.45)	1.32 (1.15, 1.52)
Formal schooling	1,579 (50)	43,484 (70)	1.00	1.00
Parity	3,154	62,320		
0	1,079 (34)	20,363 (33)	1.36 (1.25, 1.47)	1.33 (1.21, 1.45)
1-2	1,053 (33)	26,247 (42)	1.00	1.00
≥3	1,022 (32)	15,710 (25)	1.27 (1.16, 1.39)	1.26 (1.13, 1.40)
Antenatal care	3,147	62,480		
Any	2,906 (92)	60,592 (97)	1.00	1.00
None	241 (8)	1,888 (3)	1.49 (1.28, 1.73)	1.35 (1.15, 1.58)
Delivery attendant	3,166	62,726		
Physician	1,236 (39.0)	22,622 (36.1)	1.00	1.00
Nurse/Midwife	861 (27.2)	19,963 (31.8)	0.72 (0.62, 0.83)	0.66 (0.57, 0.77)
No skilled birth attendant	1,069 (33.8)	20,141 (32.1)	0.79 (0.67, 0.95)	0.58 (0.41, 0.80)
Delivery location	3,176	62,724		
Facility	2,108 (66)	42,687 (68)	1.00	1.00
Home/Other	1,068 (34)	20,037 (32)	0.96 (0.83, 1.12)	1.05 (0.77, 1.44)
Infant gender	2,934	62,720		
Male	1,677 (57)	32,406 (52)	1.22 (1.13, 1.32)	1.21 (1.11, 1.31)
Female	1,257 (43)	30,314 (48)	1.00	1.00
Daily smoking in household	3,175	62,721		
Yes	888 (28)	14,547 (23)	1.02 (0.92, 1.13)	
No	2,287 (72)	48,174 (77)	1.00	

Table 3 has the adjusted odds ratios (aOR) estimated using a multinomial logistic regression model. It shows the risk factors for the multi-level mortality variable (macerated and non-macerated stillbirths, very early neonatal deaths on days 0–2 (NMR_0-2) and later neonatal deaths days 3–28 (NMR_3-28) vs. alive on day 29). The aOR for having a macerated stillbirth in mothers exposed to HAP during pregnancy versus not exposed to HAP was 1.66 (95 % CI 1.23, 2.25). The corresponding aOR for having a non-macerated stillbirth was 1.43 (95 % CI 1.15, 1.85), very early neonatal mortality (NMR_0-2) was 1.82 (95 % CI 1.47, 2.22) and later neonatal mortality (NMR_3-28) was 1.28 (95 % CI 0.91, 1.76). Risk factors for having a macerated or non-macerated stillbirth or very early neonatal mortality (NMR_0-2), also adjusted for Global Network site, included cooking with polluting fuel, nulliparity and 3 or more prior births. Delivery by nurse or midwife was associated with lower perinatal mortality compared with delivery by a physician. Lack of formal schooling was associated with having both a macerated and non-macerated stillbirth and surprisingly, lack of antenatal care was only associated with having a non-macerated stillbirth. Male gender was associated with very early neonatal mortality (NMR_0-2) and later neonatal mortality (NMR_3-28), and nulliparity was also associated with later neonatal mortality. Presence of anyone in the household who smoked on a daily basis was not associated with having a still birth or neonatal death and was not included in the multivariate model.

**Table 3:** Risk factors for the multilevel mortality variable

Characteristic	Multivariate analysis - adjusted odds ratios (95 % Confidence Interval)				
	Macerated stillbirths	Non-Macerated stillbirths	Early neonatal mortality day 0–2 of life (NMR_0-2)	Later neonatal mortality day 3–28 of life (NMR_3-28)	Alive on day 29 (Reference Group)
HAP exposure					
Polluting fuel	1.66 (1.23, 2.25)	1.43 (1.15, 1.85)	1.82 (1.47, 2.22)	1.28 (0.91, 1.76)	1.00
Clean fuel	1.00	1.00	1.00	1.00	1.00
Maternal age					
<20	0.83 (0.55, 1.13)	1.01 (0.81, 1.24)	1.21 (0.97, 1.48)	1.05 (0.78, 1.42)	1.00
≥20	1.00	1.00	1.00	1.00	1.00
Maternal education					
No formal schooling	1.71 (1.32, 2.22)	1.51 (1.26, 1.81)	1.14 (0.90, 1.44)	1.19 (0.95, 1.47)	1.00
Formal schooling	1.00	1.00	1.00	1.00	1.00
Parity					
0	1.27 (1.03, 1.59)	1.34 (1.13, 1.56)	1.24 (1.05, 1.47)	1.43 (1.17, 1.73)	1.00
1-2	1.00	1.00	1.00	1.00	1.00
≥3	1.30 (1.01, 1.68)	1.39 (1.19, 1.65)	1.22 (1.04, 1.43)	1.11 (0.86, 1.41)	1.00
Antenatal care					
Any	1.00	1.00	1.00	1.00	1.00
None	1.41 (0.99, 2.12)	1.62 (1.23, 2.13)	0.90 (0.68, 1.21)	1.32 (0.85, 1.81)	1.00
Delivery attendant					
Physician	1.00	1.00	1.00	1.00	1.00
Nurse/Midwife	0.69 (0.52, 0.87)	0.65 (0.51, 0.82)	0.68 (0.54, 0.85)	0.87 (0.70, 1.08)	1.00
No skilled birth attendant	0.58 (0.25, 1.03)	0.50 (0.30, 0.80)	0.67 (0.39, 1.00)	0.91 (0.49, 1.67)	1.00
Delivery location					
Facility	1.00	1.00	1.00	1.00	1.00
Home/Other	0.94 (0.49, 2.30)	1.06 (0.70, 1.68)	1.00 (0.72, 1.71)	0.86 (0.48, 1.48)	1.00
Infant gender					
Male	1.08 (0.91, 1.30)	1.13 (0.99, 1.29)	1.39 (1.19, 1.60)	1.18 (1.00, 1.40)	1.00
Female	1.00	1.00	1.00	1.00	1.00

## Discussion

This study shows that in rural populations in five low resource countries, household use of polluting fuels for cooking increases the overall risk of perinatal mortality, after adjusting for maternal education, parity, antenatal visits, delivery location and attendant, and male gender. The important new finding of this research is that when the perinatal period of risk is divided into pre-partum (risk of having a macerated stillbirth), intrapartum (risk of having a non-macerated stillbirth) and postpartum (risk of neonatal death on day 0–2, 3–28 of life), exposure to HAP is associated with both types of stillbirths and early neonatal death through day 2 of life, not later neonatal death. These time periods were chosen for the secondary exploratory analyses (rather than early (day 0–7) and late (day 8–28) neonatal deaths) because there are few deaths after day 2 of life and we wanted to focus on the high mortality period between day 0 and 2 of life. Recognition of the risk of having a macerated fetus after week 20 of gestation is also important, as this outcome is not always recorded and may lead to an underestimate of the impact of exposure to HAP on adverse pregnancy outcomes.

Daily smoking in the household was not an independent predictor of perinatal mortality. It is possible that daily smoking was not an independent predictor of adverse pregnancy and neonatal outcome in our study because the exposure for most pregnant women in the rural communities studied was likely second hand smoke and exposure of the fetus and young infant to pollutants from second hand smoke would be much lower than the exposure to pollutants from household use of polluting fuel, although both second hand smoke and exposure to HAP has been associated with poor pregnancy outcomes in a prior Global Network study [23]. These different results may have been due to the specific DHS questions that were asked in this study, while the prior Global Network study asked questions adapted from the Global Youth Tobacco Survey, the 2000 US National Health Interview Survey and the Smoke-free Families Screening form. The DHS questions did not include maternal use of smokeless tobacco, which could be a risk factor for stillbirths or other adverse pregnancy outcomes. This is a limitation of our study. In future studies, it will be important to measure urine cotinine to assess actual exposure to tobacco smoke and smokeless tobacco.

Our data on the other independent risk factors for fetal or neonatal death are similar to those reported by others including no formal schooling, nulliparity and 3 or more prior births and no antenatal care visits [24, 25]. Male gender is well recognized as a risk factor particularly for neonatal mortality, [26] associated with the biological survival advantage of girls in the neonatal period. Physician assisted deliveries of women from rural communities is often due to referral of women with high risk conditions to a higher level of care. So the reduced risk of perinatal mortality associated with non-physician delivery attendants may be due to more complicated deliveries being done by physicians.

A major limitation of studies included in Pope et al’s meta-analysis [18] is the lack of a clear definition of stillbirth. A strength of this study is the accurate and complete recording of stillbirths (macerated and non-macerated) and timing of neonatal mortality by trained health care workers. However, our study has several limitations. Firstly, almost all published studies examining the effect of HAP on perinatal mortality [24, 27–31], including ours, have used the type of fuel used for cooking as a proxy for exposure to HAP. We focused on fuels used for cooking, as fuels are rarely used for heating in our Global Network sites, and did not adjust for whether cooking occurred inside or outside the household because where the cooking occurred was confounded by Global Network site. There is also some variation with the way household air pollution is categorized in prior studies, although most compare the relatively homogeneous group of clean fuels with the heterogeneous group of polluting fuels, as we did. Risk of perinatal mortality likely varies by fuel type, in part because pollutants vary by fuel type e.g., kerosene smoke pollutants are quite different from wood smoke pollutants [24]. Unfortunately, we could not analyze data on kerosene separately because only 1 % of our households used kerosene. We would have also preferred to analyse data on wood smoke and coal/charcoal as additional separate categories, but use of wood as a cooking fuel was confounded with global network location and only 3 % of households used coal/charcoal as a cooking fuel. In future studies, it will be important to measure particulate matter and other pollutants associated with biomass fuels. Secondly, we were only able to control for variables collected for the MNH Registry and specifically, we were not able to adequately control for socioeconomic status, which can be associated with pregnancy outcomes. Biomass fuels, especially fire wood collected from the forests, are used in impoverished rural homes because it is readily available and cheap [32]. The measure of socioeconomic status for this study was level of education and antenatal visits, while we did control for these proxy variables, residual confounding is possible. Although the Global Network has attempted to obtain details of maternal health before and during pregnancy (e.g., pre-pregnancy body mass index), there are limitations to the validity of these data and we are unable to address the impact of maternal conditions and BMI on pregnancy outcomes. Similarly, we do not have valid information on the neonate’s nutritional status and cannot address the effect of this confounder on perinatal mortality. Finally, we recognize that since information on exposure to cooking fuels and smoking and confounding variables were collected on day 42 postpartum, there is a potential for recall bias, which is an additional limitation.

The association between polluting fuel and perinatal mortality mediated by LBW is biologically plausible based on studies of the effects of tobacco smoking, outdoor air pollution and animal studies, but the precise mechanisms by which the varying types of HAP cause perinatal mortality and LBW is not clear. We examined the possibility of LBW being a mediator on the causal pathway between HAP and mortality using mediation analysis as follows. We assumed a causal pathway between HAP and PMR and tested to determine whether LBW contributes to the increased PMR in HAP households. In order to do this, we regressed HAP on LBW controlling for site (RR = 1.17 (1.10, 1.24), *p* < 0.0001); we then regressed HAP on PMR while controlling for LBW. The RR for HAP in the model that controls for LBW was attenuated by about 9 % (1.34 to 1.22). If LBW were the only variable on the causal pathway, then introducing LBW into the model with HAP and PMR would explain all of the variability in PMR, LBW would be a significant predictor and HAP would become non-significant in the model. Since the RR is attenuated but still significant, it suggests that some of the increased PMR in HAP households is due to LBW associated with HAP. The mediation effect of LBW on the causal pathway was further tested with Sobel’s test. The value of Sobel’s z- statistic was 5.08, *p* < 0.0001 confirming that LBW was a mediator in the causal pathway between HAP and PMR. Although the statistical analysis suggests that LBW is probably a mediator on the causal pathway, we do not have any data collected in this study to confirm or deny this. Similarly, preterm, birth defects, and maternal and neonatal complications might be on the causal pathway between exposure to HAP and mortality, or could be confounders. The statistical modelling technique to determine whether a variable is a confounder or a mediator is the same. Since we did not have any data to confirm whether it was one or the other, we decided that it would be better to exclude these variables from the model. Future research should focus on collecting data that will help to clarify these relationships.

Polyaromatic hydrocarbons (PAH) can cross the placenta and reach fetal organs [33–43]. These compounds may interfere with placental development and nutrient and oxygen delivery to the fetus [44, 45]. DNA-adduct levels of PAH in cord blood leukocytes have been linked with decreased birth weight, length, and head circumferences [46, 47]. PAH, metals, and related compounds can induce the production of cytotoxic reactive oxygen species and, ultimately, inflammatory and oxidant stress responses [48, 49]. Ultrafine particles are potent inducers of cellular heme oxygenase-1 expression and deplete intracellular glutathione, both also important in oxidant stress responses [50]. Stress responses and proinflammatory cytokines may also trigger preterm birth [51, 52], although at the maternal fetal interface, not systemically [53]. Carbon monoxide from combustion of any biomass and fossil fuels have been linked with intrauterine growth restriction, possibly as a result of carboxyhemoglobin limiting oxygen delivery to fetal tissue [54]. Early and late neonatal mortality may be caused by neonatal pneumonia.

## Conclusions

In September 2010, the United Nations Foundation announced the Global Alliance for Clean Cook Stoves, a new public-private partnership to save lives, empower women, improve livelihoods, and combat climate change by creating a thriving global market for clean and efficient household cooking solutions. The Alliance’s ‘100 by 20’ goal calls for 100 million homes to adopt clean, efficient stoves and fuels by 2020. Already there is great urgency to implement improved stoves and fuels and so there is imminent need to evaluate whether these new stoves actually do save lives [12]. Our study documents that in rural locations in five low and low-middle income countries where use of polluting fuel is widespread, large numbers of pregnant women will need to be studied to determine whether improved cook-stoves and fuels that reduce exposure to HAP also reduces perinatal mortality, LBW and neonatal mortality.

## Abbreviations

aOR: Adjusted odds ratio
aRR: Adjusted relative risk
CI: Confidence interval
DHS: Demographic Health Survey
GN: Global Network
HAP: Household air pollution
LBW: Low birth weight
MDG4: Millennium Development Goal #4
MNH: Maternal Newborn Health
NICHD: Eunice Kennedy Shriver National Institute of Child Health and Human Development
NMR_0-2: Very early neonatal mortality day 0–2
NMR_0-7: Early neonatal mortality day 0–7
NMR_3-28: Later neonatal mortality day 3–28
OR: Odds ratio
PAH: Polyaromatic hydrocarbons
PMR: Perinatal mortality rate – deaths from week 20 of gestation through day 7 of life
RR: Relative risk
